# Supplementary material for: Patterns of Intron Gain and Loss in Fungi
Source: PLoS Biol. 2004 Nov 30;2(12):e422. doi: 10.1371/journal.pbio.0020422 (PMC532390; doi:10.1371/journal.pbio.0020422)
Supplement: Table S1 — Also available at http://genes.mit.edu/NielsenEtAl/. (4.3 MB ZIP). [file pbio.0020422.st001.zip › NielsenEtAl/html/61.html]

AN8698.1.NCU01918.1.MG02971.1.FG08919.1


```
 CLUSTAL W (1.82) Multiple Sequence Alignments - Introns Inserted


Sequence 1: MG02971.1	151 aa
Sequence 2: FG08919.1	171 aa
Sequence 3: NCU01918.1	169 aa
Sequence 4: AN8698.1	170 aa
Alignment Length: 179 aa
Number Identitical Residues: 128 aa
Alignment Score (without introns) 5060


MG02971.1 	-~--------------------MSNFASQASERHNVPEIEAQTSPEVLLTPLTIARNENE
NCU01918.1	M0SQSLRPYLQAVRSSLTAALCLSNFASQTAERHNVPEVEARTSPEVLLTPLTIARNENE
FG08919.1 	-~-------MQCVRSSLTAALTLSNFASQTAERHNVPEIEAQTSPEVLLQPLTIARNENE
AN8698.1  	-~---MRPYLQCVRSSLTAALAISNFASQTSERHNVPEIEAASSPELLLNPLTISRNENE
          	       .  ..  :: :::  :******::*******:** :***:** ****:*****

MG02971.1 	RVLIEPSVNSVRISIKIKQADEIEHI~LVHKFTRFLTQRAESFFILRRKPIK0GYDISFL
NCU01918.1	RVLIEPSINSIRISIKIKQADEIEHI~LVHKFTRFLTQRAESFFILRRKPIK0GYDISFL
FG08919.1 	RVLIEPSINSVRISIKIKQADEIEHI~LVHKFTRFLTQRAESFFILRRKPIK0GYDISFL
AN8698.1  	KVLIEPSVNSVRVSIRIKQADEIEHI1LVHKFERFLTQRAESFFILRRKPVK0GYDISFL
          	:******:**:*:**:********** ***** *****************:* *******

MG02971.1 	ITNFHTEEMLKHKLVDFIIQFMEEVDKEISEMKLF0LNARARFVAESFLTP~VCVPL---
NCU01918.1	ITNFHTEEMLKHKLVDFIIQFMEEVDKEISEMKLF0LNARARFVAESFLTP0FD------
FG08919.1 	ITNFHTEEMLKHKLVDFIIQFMEEVDKEISEMKLF0LNARARFVAESFLTP~VRTNLNDT
AN8698.1  	ITNFHTEAMLKHKLVDFILQFMEEVDKEISEMKLF0LNARARFVAESFLTP~VSSVAQR-
          	******* **********:**************** *************** .    .  

MG02971.1 	----
NCU01918.1	----
FG08919.1 	VGRY
AN8698.1  	----
          	
```
